# Neuroprotective effects of ginsenosides Rh_1 _and Rg_2 _on neuronal cells

**DOI:** 10.1186/1749-8546-6-19

**Published:** 2011-05-19

**Authors:** Xiao-Fan Li, Cathy Nga-Ping Lui, Zhi-Hong Jiang, Yung Kin-Lam Ken

**Affiliations:** 1 Department of Biology, Faculty of Science, Hong Kong Baptist University, Kowloon Tong, Hong Kong SAR, China; 2 School of Chinese Medicine, Hong Kong Baptist University, Kowloon Tong, Hong Kong SAR, China

## Abstract

**Background:**

The present study investigates the effects of ginsenosides Rh_1 _and Rg_2 _against 6-hydroxydopamine (6-OHDA), a neurotoxin on SH-SY5Y cells and PC-12 cells. The effects of these two ginsenosides on neuronal differentiation are also examined.

**Methods:**

LDH assay was used to measure cell viability after exposure to 6-OHDA and ginsenosides. Neuronal differentiation was evaluated by changes in cell morphology and density of neurite outgrowths. Western blotting was used to determine the ginsenosides' effects on activation of extracellular signal-regulated protein kinases (ERKs).

**Results:**

Rh_1 _and Rg_2 _attenuated 6-OHDA toxicity in SH-SY5Y cells and induced neurite outgrowths in PC-12 cells. 6-OHDA-induced ERK phosphorylation was decreased by Rh_1 _and Rg_2_. 20(R)-form and 20(S)-form of the ginsenosides exerted similar effects in inducing neurite outgrowths in PC-12 cells.

**Conclusion:**

The present study demonstrates neuroprotective effects of ginsenosides Rh_1 _and Rg_2 _on neuronal cell lines. These results suggest potential Chinese medicine treatment for neurodegenerative disorders (*eg *Parkinson's disease).

## Background

Parkinson's disease (PD) is a common motor system disorder characterized clinically by rigidity, resting tremor and slow movements [1]. It is associated with a progressive loss of dopaminergic neurons within the *substantia nigra *and depletion of dopamine in the striatal region [2,3]. Dopamine (DA) is a catecholamine neurotransmitter in the brain, produced mainly in the *substantia nigra *and the ventral tegmental area. Six-hydroxydopamine (6-OHDA) is a hydroxylated analogue of DA. Metabolism of dopamine leads to the generation 6-OHDA [4,5] which exerts specific neurotoxicity on catecholaminergic neurons by a selective transport mechanism, including its uptake and accumulation in those neurons [6] due to its structural similarity with DA. Recent studies demonstrated that 6-OHDA toxicity might involve an extracellular autoxidation process [6,7]. Alterations in intracellular signaling pathways including the MAPKs pathway were recently found to accompany 6-OHDA toxicity. Specifically, extracellular signal-regulated protein kinases (ERK) activation and c-jun N-terminal kinase (JNK) activation have been observed in various models [8-10].

Ginseng, the fleshy root of the *Panax *species in the family Araliaceae, is an herbal medicine traditionally used in East Asia and is now popular worldwide. Recent Studies have demonstrated its beneficial effects *in vivo *and *in vitro *in various pathological conditions such as cardiovascular diseases, immunodeficiency, cancer and hepatotoxicity [11]. Moreover, increasing evidence suggests that ginsenosides are responsible for the pharmacological effects of ginseng [12]. As ginsenosides (or ginseng saponins) possess antioxidant, anti-apoptotic, anti-inflammatory and immunostimulant properties; they can positively affect neurodegenerative diseases or delay neuronal aging [11]. In fact, ginsenosides have been reported to have various actions on the central nervous system (CNS) [13,14], in particular, their anti-Parkinson effects. Ginsenosides Rb_1 _and Rg_1 _protect dopaminergic neurons *in vivo *and *in vitro *against toxicity induced by MPTP, 6-OHDA or glutamate [15-20]. They also enhance neurite outgrowth with or without stimulation of the nerve growth factor (NGF) [14,18,21]. Ginsenosides are classified into two major groups, namely dammarane and oleanane types [22]. Most ginsenosides belong to the dammarane type which is further divided into the protopanaxadiol (PPD) group and the protopanaxatriol (PPT) group according to their genuine aglycones [23]. Both ginsenosides Rh_1 _and Rg_2 _belong to the PPT group. While ginsenosides in the PPT group have generally stimulating effects on the CNS, such as anti-fatigue and hypertensive effects, anabolic stimulation, enhanced mental acuity and intellectual performance, ginsenosides in the PPD group are generally CNS-depressants with anti-stress, antipyretic and hypotensive effects [24]. However, the action mechanism of ginsenosides, Rh_1 _and Rg_2 _in particular, is still unclear. Each ginsenoside has 20(R) and 20(S) forms. However, the C-20 stereocytochemistry is relevant to the effects of ginsenosides still await investigation.

Nuclear receptors are transcriptional factors that specifically regulate target gene expression in response to hormones and other metabolic ligands [25]. Estrogen receptors (ERs), thyroid hormone receptor (TR), glucocorticoid receptors (GRs) are well-known subfamilies of nuclear receptors. The two ER subtypes, namely ERα and ERβ, together with their splice variants mediate diverse physiological processes in different tissues [26,27] while ERα seems to be the major component in mediating neuroprotection and estrogen-induced differentiating effects [28,29]. Previous studies revealed that liganded ERα enhanced NGF-induced differentiation in PC-12 cells while in the absence of 17β-estradiol (17βE2), the expression of ERα actually partly suppressed NGF-induced neurite outgrowth or expression of neuronal markers [30]. Increased NGF-induced gene expression by 17βE2 suggests the transcriptional activity of ERα on PC-12 cell differentiation. By contrast, several studies demonstrated that ERα was involved in the mediation of neuronal survival against various insulted including glutathione depletion, serum deprivation and glutamate toxicity [29,31,32].

Mitogen-activated protein kinases (MAPKs) are an evolutionarily conserved family of serine/threonine-specific kinases that regulate various cellular activities, such as cell proliferation, differentiation and apoptosis [33,34]. In mammals, MAPKs include the ERKs, p38 MAPK and c-Jun NH_2_-terminal kinases (JNKs) [35]. ERK's role in neurotoxicity is dependent on the experimental paradigm. Previous studies suggested that the activation of ERK by growth factors or by stress conferred a survival advantage to cells [36,37]; however, recent studies found that ERK promoted neuronal cell death *in vivo *and *in vitro *[38,39] while inhibition of ERK had protective effects in various models of neuronal cell death [40-42].

The present study aims to evaluate the effects of ginsenosides Rh_1 _and Rg_2 _on neuroprotection, cell differentiation and on ERK activation in neuronal cells.

## Methods

### Chemicals

**Figure 1:**
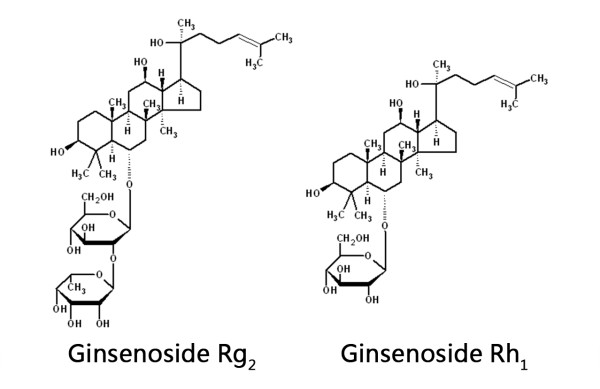
**Chemical structure of ginsenosides Rg_2 _and Rh_1_**.

Ginsenosides Rh_1 _and Rg_2 _(enantiomeric mixtures) as well as individual stereoisomers, *ie *20(R)-Rh_1_, 20(S)-Rh_1_, 20(R)-Rg_2 _and 20(S)-Rg_2 _in powder form (>99% purity) were provided by ZHJ (Figure 1). The powder was dissolved in dimethyl sulfoxide (DMSO) to a stock solution of 10 mM. Further dilution was made in complete culture medium or serum-free medium, depending on the experimental setup.

Nerve Growth Factor-β (NGF-β) from rat (Sigma-Aldrich; USA) was reconstituted using sterile PBS containing 0.1% BSA to a stock concentration of 1 μg/ml. Further dilution was made in complete culture medium or serum-free medium, depending on the experimental setup.

Six-hydroxydopamine (6-OHDA) hydrochloride (Sigma) was dissolved in sterile Hank's Buffered Salt Solution (HBSS) containing 0.1% ascorbic acid to a 1 mM stock solution, and further dilution to target concentrations was made in serum-free medium.

### Cell culture

SH-SY5Y cells were cultured in Dulbecco's modified eagle medium containing nutrient mixture F-12 (DMEM/F12) (Gibco; USA) with 10% Fetal Bovine Serum (FBS) (Gibco; USA) and 0.5% Penicillin-Streptomycin-Neomycin (PSN) Antibiotic Mixture (Gibco; USA). The cells were incubated in a humidified incubator at 37°C, 5% CO_2_. The culture medium was renewed every three to four days and the cells were subcultured every seven to eight days. The cells were detached from the culture flask by treatment with trypsin-EDTA (Gibco; USA) at a ratio of 1 ml per 25 cm^2 ^for half a minute.

PC-12 cells were cultured in F-12 K Medium (Gibco; USA) with 15% Horse Serum (HS) (Gibco; USA), 2.5% FBS (Gibco; USA) and 1% PSN Antibiotic Mixture (Gibco). The cells were seeded on Type-I rat-tail collagen (Millipore; USA) coated culture flasks (Nunclon; USA), 6-well plastic plates (Iwaki; Japan) and 4-well plastic plates (Nunclon; USA). The cells were incubated in a humidified incubator at 37°C, 5% CO_2_. The culture medium was renewed every three to four days and the cells were subcultured every seven to eight days. The cells were detached by physical flushing.

### Neurite outgrowth assessments

PC-12 cells were seeded in 4-well plates at a density of 30,000 cells per well in complete culture medium. The medium was changed after 24 hours to complete the culture medium plus 20 μM ginsenoside Rh_1 _or Rg_2 _with or without 5 ng/ml NGF co-treatment. The concentration of NGF was chosen based on previous observations that 5 to 10 ng/ml NGF-β in serum-free medium induced optimal neurite outgrowth in PC-12 cells [26]. After 48 hours, the cells were observed under an inverted light microscope (ZEISS; Germary) at 200 × magnification and photos were taken for subsequent quantification of neurite outgrowth.

The cells were classified according to their morphology into three groups [43], namely (1) cells with long neuritis (*ie *cells with at least one neurite twice the length of its cell body diameter); (2) cells with short neuritis (*ie *cells without a long neurite but with at least one neurite that was longer than its cell body diameter); (3) cells without neuritis (*ie *cells without any neurite outgrowth that was longer than its cell body diameter. At least 120 cells were counted for each treatment. The percentages of each group of cells in each treatment were determined.

### Analysis of cytotoxicity

Cytotoxicity after 6-OHDA and/or ginsenosides exposure was quantitatively measured by LDH cytotoxicity assay with Cytotoxicity Detection Kit (Roche Applied Science; Germary). The cells were seeded in 96-well plates at a density of 30,000 cells per well. For 6-OHDA and ginsenosides toxicity assay, 24 hours after seeding, the cells were washed once with serum-free medium, and then treated with different concentrations of 6-OHDA (5, 10, 20, 50 and 100 μM) or ginsenosides (10 and 20 μM of Rh_1 _or Rg_2_) for another 24 hours. Low control (serum-free medium) and high control (serum-free medium containing 2% Triton X-100) groups were set up to represent normal cell death and maximum cell death respectively. For the assay for ginsenosides' effects on 6-OHDA toxicity, 24 hours after seeding, the cells were pre-incubated in serum-free medium containing ginsenosides (10 and 20 μM of Rh_1 _or Rg_2_) for 24 hours. Then the cells were challenged with 6-OHDA (40 or 60 μM) with or without ginsenosides co-treatment for another 24 hours.

Prior to LDH assay, the 96-well plates were centrifuged (Beckman Allegra 6R; Beckman Instruments, USA) at 1000 g for 10 minutes to sediment the cells. Then 46 μl of supernatant was drawn from each well to a new empty well. The dye solution was mixed with the catalyst solution at a volume ratio of 45:1 and immediately after, 46 μl of reaction mixture was added to each well. The plate was incubated in the dark for 30 minutes, and then the optical density of the reaction mixture was measured with a multi-functional plate reader (Tecan Infinit F200; TECAN; Switzerland) at 495 nm with reference at 690 nm. The readings were normalized by subtracting the optical density of corresponding medium. The percentage of cell death (cytotoxicity) was calculated according to the following formula:

### Western blot analysis of ERK1/2 activation

The cells were seeded in 6-well plates at a density of 1,000,000 cells per well in complete culture medium. For SH-SY5Y cells, treatment was applied 24 hours after seeding whereas for PC-12 cells, 24 hours after seeding the medium was changed to complete medium supplemented with 5 ng/ml NGF for 48 hours to induce differentiation. Treatment was done with serum-free medium for both cells. The cells were exposed to 20 μM ginsenoside for 24 hours and then 20 μM ginsenoside plus 50 μM 6-OHDA for 3 hours. The cells were washed by ice-cold PBS before lysed with lysis buffer containing Protein Extraction Reagent (Novagen; USA) and Protease Inhibitor Cocktail Set III (Calbiochem; USA) (200:1). The cell lysate was collected and centrifuged (5430R; Eppendorf; Germany) (14,000*g*,) at 4°C for 30 minutes. The supernatant containing the proteins was collected for protein quantification or storage at -80°C.

The protein concentration in the lysate was determined with a commercially available kit (Bio-Rad; USA) and calculated from a standard protein concentration curve. Protein samples were adjusted to equal concentration and volume by lysis buffer and then mixed with equal volume of sampler buffer (Bio-Rad; USA) containing 5% β-mercaptoethanol by volume. The protein samples were heated at 100°C for five minutes before electrophoresis. The proteins were separated on SDS-polyacrylamide gel (4.5% stacking gel, 10% lower gel) and then transferred to Polyvinylidene Fluoride (PVDF) Membrane (Bio-Rad; USA) overnight. The membrane was blocked with 5% non-fat dry milk in Tris buffered saline-Tween (TBST) solution. The membrane was then incubated with Phospho-p44/42 MAPK (Erk1/2) or p44/42 MAPK (Erk1/2) antibody for two hours followed by horseradish peroxidase (HRP)-conjugated secondary antibody for one hour. Bands on the PVDF membranes were visualized by a commercially available enhanced luminal-based chemiluminescent substrate (WESTSAVE Up^TM^; AbFrontier; Korea) and developed on films (Agfa; Germary). The integrated optical density (IOD) of bands was measured with Metamorph software (Universal Imaging Corporation; USA).

### Statistical analysis

All data were presented as mean ± standard deviation (SD) unless otherwise indicated. Statistical differences between the treatment and control groups were analyzed by Welch's t-test with SigmaPlot 11.0 software (Systat Software, Inc.; Canada). For comparison between multiple groups, one way analysis of variance (ANOVA) was used followed by a Dunnett's *post-hoc *test. *P *< 0.05 was considered statistically significant.

## Results

### 6-OHDA and ginsenosides cytotoxicity

**Figure 2:**
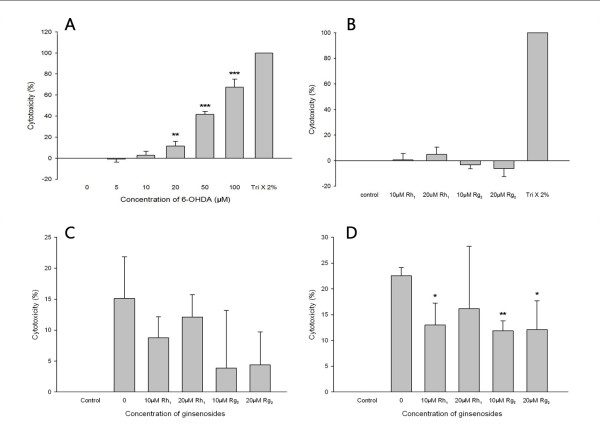
**Figures showing the effect of ginsenoside treatments on SH-SY5Y cells against 6-OHDA toxicity **a. Six-hydroxydopamine toxicity on SH-SY5Y cells. The percentage of cell death (cytotoxicity) after 24 hours of exposure to different concentrations of 6-OHDA. Values are presented as mean ± SD (*n *= 3). (Welch's t-test, ** *P *= 0.010, ***P < 0.001, vs. control). Negative percentage is considered to be zero percentage as it is resulted by calculation of the LDH assay formula. b. Ginsenosides toxicity on SH-SY5Y cells. The percentage of cell death (cytotoxicity) after 24 hours of exposure to different concentrations of ginsenosides Rh_1 _and Rg_2 _Values are presented as mean ± SD (*n *= 3). The cytotoxicity of ginsenoside-treated groups and the control group was not significantly different (one way ANOVA, *P *= 0.110). c. Effect of ginsenoside pretreatment on 40 μM 6-OHDA toxicity on SH-SY5Y cells. The percentage of cell death (cytotoxicity) after 24 hours of pretreatment of ginsenosides Rh_1 _and Rg_2 _(10 μM and 20 μM) followed by 24 hours co-treatment with ginsenosides together with 40 μM 6-OHDA. Values are presented as mean ± SD (*n *= 3). The cytotoxicity of ginsenoside-pretreated groups were not significantly different from that of the un-pretreated group (one way ANOVA, *P *= 0.184). d. Effect of ginsenoside pretreatment on 60 μM 6-OHDA toxicity on SH-SY5Y cells. The percentage of cell death (cytotoxicity) after 24 hours of pretreatment of ginsenosides Rh_1 _and Rg_2 _(10 μM and 20 μM) followed by 24 hours co-treatment with ginsenosides together with 60 μM 6-OHDA. Values are presented as mean ± SD (*n *= 3). (Welch's t-test, *P < 0.05, ** *P *< 0.01, vs. un-pretreated group. 10 μM Rh_1_: *P *= 0.022; 10 μM Rg_2_: *P *= 0.002; 20 μM Rg_2_: *P *= 0.036).

Cytotoxicity of 6-OHDA and ginsenosides Rh_1 _and Rg_2 _on SH-SY5Y cells was tested with the LDH assay. A significant increase (*P *= 0.010) in LDH release was observed following 24 hours of incubation with 6-OHDA at concentrations higher than 20 μM (Figure 2a), indicating that 6-OHDA exerted toxicity on SH-SY5Y cells. It may be suggested that the percentage of cell death increased in a dose-dependent manner within the range of 5 μM to 100 μM 6-OHDA. 50% cell death was estimated to occur at approximately 60 μM 6-OHDA (LC-50). Based on this experiment, two concentrations (40 μM and 60 μM) around and lower than the LC-50 were chosen for later experiments examining the effects of ginsenoside pretreatment on 6-OHDA toxicity.

No significant difference in LDH release was observed following 24 hours of incubation with the two ginsenosides (10 μM and 20 μM) comparing with the control group (Figure 2b). These two concentrations were used for subsequent experiments examining the effects of ginsenoside pretreatment on 6-OHDA toxicity.

### Effects of ginsenoside pretreatment on 6-OHDA toxicity

A decrease in mean cytotoxicity was observed for ginsenoside-pretreated groups upon exposure to both 40 and 60 μM 6-OHDA. Statistical analysis showed that upon 40 μM 6-OHDA exposure, the mean toxicity for ginsenoside-pretreated groups were not significantly different (*P *= 0.184, One Way ANOVA) from that of the un-pretreated group (Figure 2c). However, upon 60 μM 6-OHDA exposure, the mean toxicity for three ginsenoside-pretreated groups (10 μM Rh_1_: 13.02 ± 4.26%; 10 μM Rg_2_: 11.86 ± 1.95%; 20 μM Rg_2_: 12.12 ± 5.57%) were found to be significantly different (*P *= 0.022 for 10 μM Rh_1 _and *P *= 0.036 for 20 μM Rg_2_; *P *= 0.002 for 10 μM Rg_2_) from that of the un-pretreated group (22.55 ± 1.61%; Figure 2d). These results suggest neuroprotective effects of ginsenosides Rh_1 _and Rg_2 _against 6-OHDA toxicity on SH-SY5Y cells.

### Neurite outgrowth assessment and morphological observation

**Figure 3:**
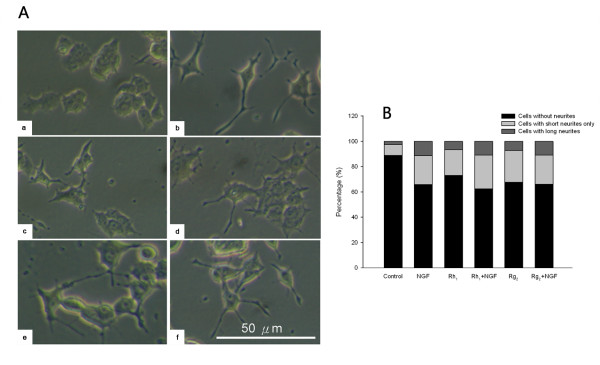
**Comparison of morphology and quantitative changes in PC-12 cells** a. Morphology comparison of PC-12 cells with or without ginsenoside and/or NGF treatment. (A) Control; (B) 5 ng/ml NGF; (C) 20 μM Rh_1_; (D) 20 μM Rh_1 _+ 5 ng/ml NGF; (E) 20 μM Rg_2_; (F) 20 μM Rg_2 _+ 5 ng/ml NGF. Scale Bar: 50 mb. Quantitative changes in PC-12 cell morphology. The stacked bars illustrate the percentages of cells that do not possess neurites, possess short neurites only, or possess long neurites in each treatment group. At least 120 cells were counted for each treatment. Ginsenosides Rh_1 _and Rg_2 _(20 μM) both increased the percentage of cells possessing short or long neurites in the absence of NGF (Rh_1_: 20.3%, 6.5%; Rg_2_: 25.1%, 7.3%) compared to the control group (8.5%, 2.6%). In the presence of NGF (5 ng/ml) the effects of Rh_1 _and Rg_2 _were mostly enhanced, but were not greatly different from NGF treatment alone (Rh_1_+NGF: 26.8%, 10.8%; Rg_2_+NGF: 22.9%, 10.9%; NGF: 22.7%, 11.3%).

The morphology of PC-12 cells was examined under inverted light microscope 48 hours after treatment. In their native states the PC-12 cells appear polygonal in shape and very few cells possess neurites while upon 5 ng/ml NGF exposure the cells extend obvious neurite outgrowths. Rh_1 _and Rg_2 _treatments both enhanced neurite outgrowths in the absence of NGF while their effects were potentiated with NGF co-treatment (Figure 3a). The morphological changes of PC12 cells were then quantified. After treatment with ginsenosides Rh_1 _and Rg_2_, the percentage of PC12 cells possessing neurites was more than that of control. (Figure 3b).

### Inhibition of 6-OHDA-induced ERK1/2 phosphorylation by ginsenosides

**Figure 4:**
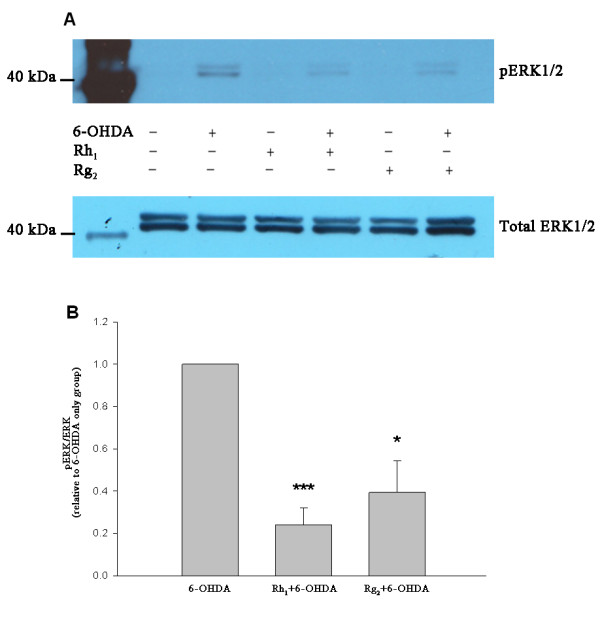
**Inhibition of ERK1/2 phosphorylation by ginsenosides Rh_1 _and Rg_2 _in SH-SY5Y cells**. (A) Representative immunoblots showing the reduction in ERK1/2 phosphorylation by ginsenosides pretreatment in SH-SY5Y cells. (B) Bar chart showing reduction in IOD_pERK_/IOD_ERK _of ginsenosides pretreated groups relative to the 6-OHDA control group (data presented as mean ± SD, *n *= 3). (Welch's t-test, * *P *= 0.015, *** *P *< 0.001).

**Figure 5:**
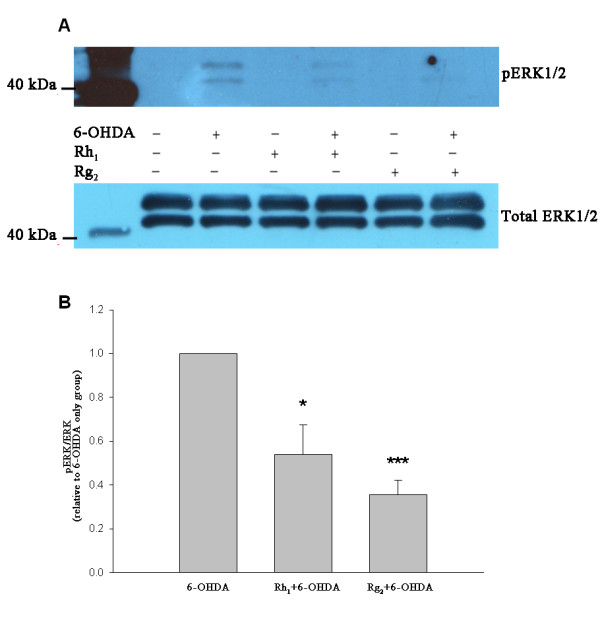
**Inhibition of ERK1/2 phosphorylation by ginsenosides Rh_1 _and Rg_2 _in PC-12 cells**. (A) Representative immunoblots showing the reduction in ERK1/2 phosphorylation by ginsenosides pretreatment in PC-12 cells. (B) Bar chart showing reduction in IOD _pERK_/IOD_ERK _of ginsenosides pretreated groups relative to the 6-OHDA control group (data presented as mean ± SD, *n *= 3). (Welch's t-test, * *P *= 0.027, *** *P *< 0.001).

50 μM 6-OHDA induced ERK1/2 phosphorylation in both SH-SY5Y cells and PC-12 cells after three hours of incubation while without 6-OHDA the phosphorylation of ERK1/2 was barely detectable. Pretreatment with ginsenosides Rh_1 _(Figure 4) or Rg_2 _(Figure 5) for 24 hours reduced the levels of ERK1/2 phosphorylation in both cells. Statistical analysis (Welch's *t*-test) showed that the means of IOD_pERK _/IOD_ERK _relative to the 6-OHDA control group were significantly reduced (SH-SY5Y *:P < 0.001 *for Rh_1 _and *P *= 0.015 for Rg_2_; PC-12: *P *= 0.027 for Rh_1 _and *P < 0.001 *for Rg_2_) with ginsenoside pretreatment (Figures 4 and 5). These results suggest a protective role of ginsenosides Rh_1 _and Rg_2 _on both cells against 6-OHDA toxicity.

### Ginsenoside stereoisomers induce neurite outgrowth

Neurite outgrowth assessment in PC12 cells was repeated with the individual stereoisomers of ginsenosides, *ie *20(R)-Rh_1_, 20(S)-Rh_1_, 20(R)-Rg_2 _and 20(S)-Rg_2_.

**Figure 6:**
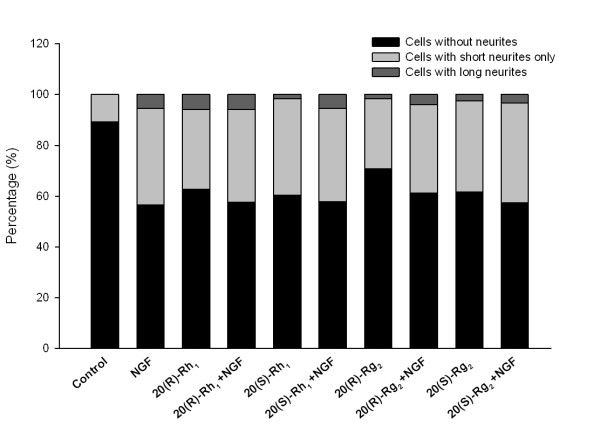
**Comparison of ginsenoside stereoisomers' effects on PC-12 cell morphology**. The stacked bars illustrate the percentages of cells that do not possess neurites, possess short neurites only, or possess long neurites in each treatment group. At least 160 cells were counted for each treatment. 20(R)-Rh_1_, 20(S)-Rh_1_, 20(R)-Rg_2 _and 20(S)-Rg_2 _(20 μM) all increased the percentage of cells possessing short or long neurites in the absence of NGF compared to the control group. In the presence of NGF (5 ng/ml) the neurite outgrowth were slightly enhanced, and no obvious difference in the effects were observed between 20(R)-ginsenosides and 20(S)-ginsenosides.

The percentage of cells possessing neuritis with the treatments of all four ginsenoside stereoisomers was found to be higher than that of control. And these treatments increased the neurite outgrowth in the absence of NGF while their effects potentiated with NGF co-treatments (Figure 6).

## Discussion

The present study demonstrates that 6-OHDA is cytotoxic to SH-SY5Y cells, and the toxicity increases in a dose-dependent manner. Pretreatment with ginsenosides Rh_1 _or Rg_2 _attenuates the 6-OHDA toxicity while not being toxic to the cells themselves. The results suggests that Rh_1 _and Rg_2 _may have induced changes in cellular activity, which helped the cells overcome 6-OHDA toxicity. It is well documented that oxidative stress is implicated in 6-OHDA-induced neuronal cell death [6,17]. The pathophysiology of many neurodegenerative disorders, including Alzheimer's disease and PD are also closely associated with oxidative damage [44]. Neuroprotection can therefore be partly achieved by counteraction of the oxidative stress with various anti-oxidants, such as glutathione, flavonoids, estrogens and phytoestrogens [44-46]. Ginsenosides have been widely reported to have anti-oxidation activities [15-17] and to promote neurite outgrowth [14,18]. A study by Liu *et al*. on the structure-activity relationship predicts that Rh_1 _is an anti-oxidant while Rg_2 _is a pro-oxidant [47]; however, Rg_2 _has been reported in other studies to have exhibited an anti-oxidation effect [46,48]. To further elucidate the mechanisms of Rh_1 _and Rg_2_, we will investigate whether anti-oxidative activity plays a role here.

The neuroprotective effects of Rh_1 _and Rg_2 _were also exemplified in MAPK/ERK signaling pathway. 6-OHDA induced ERK1/2 phosphorylation in SH-SY5Y cells as well as PC-12 cells, and the phosphorylation could be partly inhibited by pretreatment with Rh_1 _and Rg_2_. It has been reported that ginsenosides may bind to transmembrane membrane receptors to activate related signaling pathways downstream [49]. The MAPK-regulated kinases have a prominent role in regulating cellular processes such as proliferation, differentiation and adaptation [8]. Activation of two families of MAPKs, JNK/SAPK and p38 MAPK is often correlated with neurodegeneration while the role of ERKs is less clear and may vary depending on the specific cell type [45]. In the 6-OHDA neuronal models, there seems to be a time course-dependent relationship between ERK phosphorylation and its effects. The first peak of phosphorylated ERK around 15 minutes after 6-OHDA treatment appears to be pro-survival whereas the second one that comes after several hours results from sustained mitochondrial ERK phosphorylation which enhances neuronal cell death [50,51]. In the present study, significant ERK1/2 phosphorylation was found 3 hours after the 6-OHDA treatment, which is likely to be sustained rather than transient. However, we do not prelude that the change in ERK1/2 phosphorylation could be a biphasic response. The reduction of ERK1/2 phosphorylation by Rh_1 _or Rg_2 _pretreatment may indicate their neuroprotective effects against 6-OHDA toxicity. Another study also found similar inhibition effects on ERK1/2 phosphorylation exerted by Rg_1 _[8].

In the present study, wild-type PC-12 cells were used as a model for neuronal differentiation. The result showed that ginsenosides Rh_1 _and Rg_2 _induced neurite outgrowth both in the absence and presence of NGF. However, the dose-response relationship and time-dependent changes, and whether this effect promotes neuroprotection remain to be determined. The synergistic effect between NGF and ginsenosides was not apparent, perhaps because the NGF concentration used was already very potent in inducing PC-12 cell differentiation, or perhaps the incubation time was not long enough for that to occur. The mechanism of neurite induction by ginsenosides is still undefined but may be related to nuclear receptor signaling.

Ginsenosides are steroidal saponins similar to estradiol in terms of their chemical structure (Figure 1). They have a rigid four trans-ring steroid skeleton, with a modified side-chain at C20 whereas estradiol does not possess a side-chain [52]. This structural similarity may be the cause for their similar activities as well, for instance, binding to the steroid hormone receptor ERα. Moreover, ginsenosides and estrogens share many of their target tissues. Previous studies have already demonstrated estrogen-like activity of several ginsenosides, including Rg_1_, Rb_1 _and Rh_1_; however, it remains controversial as to whether or not the activation of ERα is dependent on ligand binding [49,52-55]. Nevertheless, the neuroprotective effects of estrogen also includes nongenomic mechanisms that may involve MAPK or Akt signaling, as well as its antioxidant ability, both of which may be ER-independent [56]. Thus, for the elucidation of the mechanisms of Rh_1 _and Rg_2_, further studies are warranted to test for their possible interactions with ERα (ligand binding assays; response genes expression). More investigations on ER-independent estrogen action may also contribute to our understanding of ginsenosides' estrogen-like effects.

Most ginsenosides isolated are present naturally as enantiomeric mixtures [57]. The structural factor involved is the stereochemistry at carbon-20 position. Recent studies showed that different stereoisomers of the same ginsenoside, *ie *20(R)-ginsenoside and 20(S)-ginsenoside have different pharmacological effects [58,59]. Conversely, the present study suggests that the neuroprotective properties of ginsenosides Rh_1 _and Rg_2 _may not be related to their C-20 stereochemistry. Therefore, whether C-20 stereochemistry affects ginsenoside action may vary from case to case. Further investigation may delineate the structure-function relationship of ginsenosides.

## Conclusion

6-OHDA induces cell death in SH-SY5Y cells in a dose-dependent manner while pre-incubation with ginsenosides Rh_1 _and Rg_2 _may attenuate such toxicity, possibly by anti-oxidation, activating nuclear receptors or modulations on intracellular signaling pathways. ERK1/2 phosphorylation is observed after 6-OHDA treatment in both SH-SY5Y cells and PC-12 cells. Pre-incubation with Rh_1 _or Rg_2 _reduces 6-OHDA-induced ERK1/2 phosphorylation, which is possibly neuroprotective to the cells. Rh_1 _and Rg_2 _also induce neurite outgrowth in wild type PC-12 cells both in the presence and absence of NGF. C-20 stereochemistry does not play a part in the action of the two ginsenosides but their exact mechanism of neuroprotection remains unclear.

## Abbreviations

17βE2: 17β-estradiol; 6-OHDA: 6-hydroxydopamine; JNK: c-jun N-terminal kinase; DA: Dopamine; DMEM/F12: Dulbecco's modified eagle medium containing nutrient mixture F-12; ERs: Estrogen receptors; ERKs: extracellular signal-regulated protein kinases; GRs: glucocorticoid receptors; HS: Horse Serum; MAPKs: Mitogen-activated protein kinases; NGF: nerve growth factor; PD: Parkinson's disease; PSN: Penicillin-Streptomycin-Neomycin; PPD: protopanaxadiol; PPT: protopanaxatriol; SD: Standard deviation; TR: thyroid hormone receptor;

## Competing interests

The authors declare that they have no competing interests.

## Authors' contributions

XFL and KKLY designed the study. XFL conducted the experiments, analyzed the data and drafted the manuscript. CNPL revised the manuscript. ZHJ helped conduct the experiments. All authors read and approved the final version of the manuscript.
